# Missense mutations near the N-glycosylation site of the A2 domain lead to various intracellular trafficking defects in coagulation factor VIII

**DOI:** 10.1038/srep45033

**Published:** 2017-03-22

**Authors:** Wei Wei, Chunlei Zheng, Min Zhu, Xiaofan Zhu, Renchi Yang, Saurav Misra, Bin Zhang

**Affiliations:** 1 Genomic Medicine Institute, Cleveland Clinic Lerner Research Institute, Cleveland, OH, USA; 2 State Key Laboratory of Experimental Hematology, Institute of Hematology and Blood Diseases Hospital, Chinese Academy of Medical Sciences & Peking Union Medical College, Tianjin, China; 3 Department of Pathology, Karamay Central Hospital, Karamay, Xinjiang, China; 4 Department of Biochemistry and Molecular Biophysics, Kansas State University, Manhattan, KS, USA

## Abstract

Missense mutation is the most common mutation type in hemophilia. However, the majority of missense mutations remain uncharacterized. Here we characterize how hemophilia mutations near the unused N-glycosylation site of the A2 domain (N582) of FVIII affect protein conformation and intracellular trafficking. N582 is located in the middle of a short 3_10_-helical turn (D580-S584), in which most amino acids have multiple hemophilia mutations. All 14 missense mutations found in this 3_10_-helix reduced secretion levels of the A2 domain and full-length FVIII. Secreted mutants have decreased activities relative to WT FVIII. Selected mutations also lead to partial glycosylation of N582, suggesting that rapid folding of local conformation prevents glycosylation of this site in wild-type FVIII. Protease sensitivity, stability and degradation of the A2 domain vary among mutants, and between non-glycosylated and glycosylated species of the same mutant. Most of the mutants interact with the ER chaperone BiP, while only mutants with aberrant glycosylation interact with calreticulin. Our results show that the short 3_10_-helix from D580 to S584 is critical for proper biogenesis of the A2 domain and FVIII, and reveal a range of molecular mechanisms by which FVIII missense mutations lead to moderate to severe hemophilia A.

Hemophilia A is an X-linked recessive bleeding disorder caused by a quantitative or qualitative deficiency of coagulation factor VIII (FVIII). FVIII is a cofactor for factor IXa (FIXa) that accelerates the activation of factor X, playing an essential role in the intrinsic pathway of the coagulation cascade^1^. Based on their plasma FVIII levels, hemophilia A patients are classified as mild (0.05–0.4 U/ml), moderate (0.01–0.05 U/ml) or severe (<0.01 U/ml)^2^. Patients with severe hemophilia A, who comprise approximately 60% of cases, are at particularly high risk of debilitating or even fatal bleeding. FVIII is a 2351 amino acid glycoprotein composed of six domains in the order: A1-A2-B-A3-C1-C2^3^,^4^. It is synthesized in the endoplasmic reticulum (ER) of endothelial cells^5^,^6^,^7^,^8^. In the secretory pathway, FVIII is processed into a heterodimer consisting of the heavy chain (A1, A2 and B domains) and the light chain (A3, C1 and C2 domains). During FVIII activation, the B domain is released and thrombin cleaves the heavy chain into A1 and A2 fragments to form an active heterotrimer, A1/A2/A3-C1-C2^9^.

Most proteins that pass through the eukaryotic secretory pathway are glycosylated on asparagine (N) residues of the consensus sequence N-X-S/T (X≠P) as they enter the ER^10^. One of two isoforms of the oligosaccharyltransferase (OST), STT3A or STT3B, transfers a pre-formed glycan structure onto the asparagine residue^11^. Upon glucose trimming, the oligosaccharide serves as a tag for ER chaperones to detect the folding status of proteins and ultimately ensures that export is restricted only to properly folded glycoproteins^12^. N-linked glycans are further modified in the Golgi by distinct modifying enzymes. Glycosylation also modifies properties of glycoproteins and is necessary for the stability and function of some glycoproteins^13^,^14^. FVIII is highly N-glycosylated with 25 consensus sequences within its B domain and 5 consensus sequences outside the B domain (N41, N239, N582, N1810 and N2118). The high-mannose glycans on FVIII interact with the carbohydrate recognition domain of the LMAN1 subunit of the LMAN1-MCFD2 cargo receptor complex, which mediates the export of FVIII out of the ER^15^,^16^,^17^,^18^,^19^. FVIII also interacts with the asialoglycoprotein receptor through glycans located on the B domain^20^, although how this interaction regulates FVIII export or clearance is poorly defined^21^.

Two major chaperone systems in the ER, BiP (GRP78) and calnexin/calreticulin (CNX/CRT), facilitate proper folding of proteins including FVIII^11^,^12^,^22^. These chaperone systems also regulate the degradation of misfolded proteins through ER-associated degradation (ERAD). BiP, a member of the heat shock protein 70 family, interacts with hydrophobic segments of unfolded proteins to promote protein folding and prevent aggregation. The lectins CNX and CRT are involved in folding and quality control of glycoproteins in the ER through the ‘calnexin/calreticulin cycle’. Binding of an 110-amino acid region of the FVIII A1 domain to BiP inhibits secretion of FVIII from cells^23^, while mutagenesis of a potential BiP binding site of FVIII enhances FVIII secretion^24^. Both CNX and CRT also interact with FVIII, mainly through the B domain^25^.

The most common mutations in hemophilia A patients are missense mutations, with over 1,400 such mutations reported to date (www.factorviii-db.org). Some missense mutations were reported to decrease secretion or stability of the mutant protein^26^,^27^,^28^. Several molecular mechanisms of missense mutations that produce dysfunctional FVIII have also been reported; these include decreased interaction with VWF, FIXa and phospholipids, altered cleavage by thrombin, and accelerated dissociation rate of the A2 domain^29^,^30^,^31^,^32^. However, the mechanisms of the majority of missense mutations are still unknown. In this study, we focused on a surface-exposed glycosylation consensus site within the A2 domain of FVIII, N582, that is nevertheless unglycosylated in the wild-type protein. We utilized a mammalian cell culture system to characterize the relationships between the glycosylation of N582, nearby missense mutations, and the stability, secretion and activity of both the isolated A2 domain and full-length FVIII. We determined that the local conformation of FVIII near N582 is critically tuned and that global properties of the A2 domain and FVIII are highly sensitive to changes in this region induced by hemophilia A missense mutations.

## Results

### N-linked glycosylation and secretion levels of individual domains of FVIII

**Figure 1:**
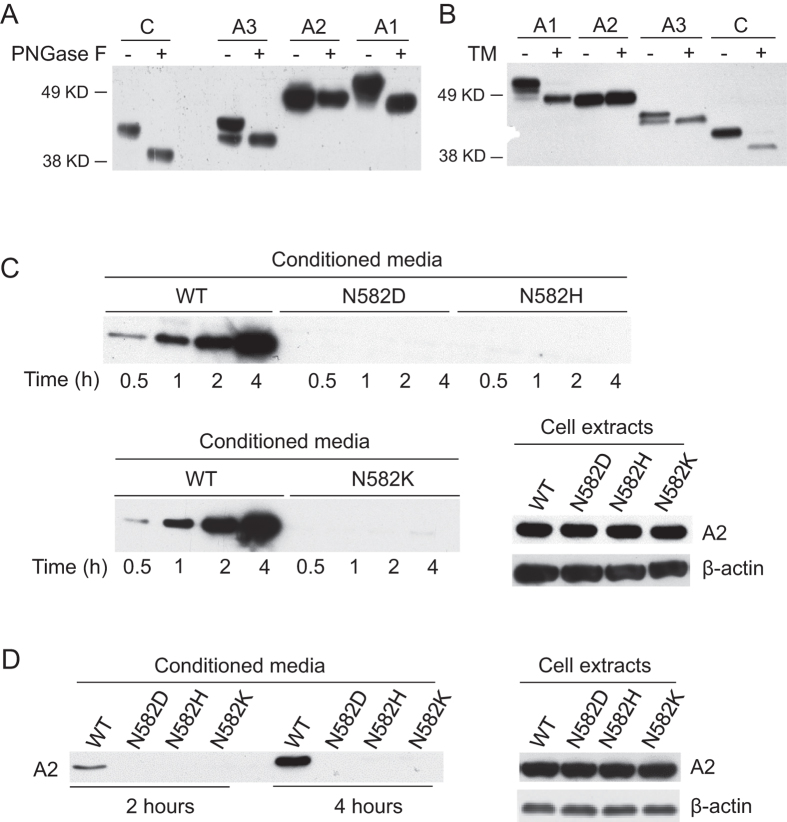
The A2 domain is not N-glycosylated and N582 mutations result in defective secretion of the A2 domain. (**A**) COS-1 cells were transfected with constructs that express Flag-tagged individual domains (A1, A2, A3 and C) of FVIII. Cell lysates were digested with PNGase F or left untreated, and subsequently immunoblotted with an anti-Flag antibody. (**B**) COS-1 cells were transfected with individual FVIII domain constructs in duplicates. One set of transfected cells were treated with 2 μg/ml tunicamycin for 12 h before lysis. (**C**) COS-1 cells were transfected with a construct expressing WT A2 domain or one of three different A2 domain mutants. A fresh medium change was carried out thirty hours after transfection. At the indicated time after the change of medium, conditioned media were collected and immunoprecipitated with a mouse monoclonal anti-Flag antibody and analyzed by immunoblotting with a rabbit anti-Flag antibody. Thirty-six hours after transfection, cells were lysed and equal amounts of cell extracts were immunoblotted with anti-Flag and anti-β-actin antibodies. (**D**) Six hours after transfection, COS-1 cells were cultured at 28 °C for 24 hours before fresh medium change. Conditioned media were collected at 2 and 4 h and secreted A2 protein was analyzed by immunoprecipitation and immunoblotting. Thirty-six hours after transfection, cells were lysed and equal amounts of cell extracts were immunoblotted with anti-Flag and anti-β-actin antibodies. All experiments were performed independently twice.

The five consensus N-linked glycosylation sequences in FVIII outside of the B domain are located in the A1 domain (N41, N239), the A2 domain (N582), the A3 domain (N1810) and the C1 domain (N2118). To investigate which consensus sites are actually glycosylated in cells, we expressed individual domains of FVIII (A1, A2, A3 and C) in both COS-1 and HEK293T cells and treated cell lysates with PNGase F to cleave N-linked glycans. After PNGase F treatment, proteins that contain N-linked glycans exhibit increased mobility in SDS-PAGE compared to untreated controls. The A1, A3, and C domains all showed increased mobility after digestion with PNGase F, but the A2 domain remained unchanged (Fig. 1A and Figure S1A). Next, we treated cells with tunicamycin, which blocks the first step of N-linked glycosylation. Twelve hours after tunicamycin treatment, COS-1 and HEK293T cells transfected with individual FVIII domain plasmids were harvested and the sizes of FVIII domains were analyzed by anti-Flag immunoblotting. Upon tunicamycin treatment, A1, A3 and C domains all shifted to increased mobility/smaller species, but the A2 domain remained unchanged (Fig. 1B and Figure S1B). These results confirmed that the A1, A3 and C domains of FVIII are N-glycosylated. In contrast, N582 in the A2 domain is not glycosylated in cells, consistent with its lack of glycosylation in recombinant FVIII^33^,^34^,^35^,^36^. Of note, we detected a minor non-glycosylated fraction of the A3 domain before PNGase F digestion and without tunicamycin treatment, showing that the A3 domain is incompletely glycosylated after translation.

### Missense mutations of N582 found in hemophilia A patients result in defective secretion of the A2 domain

Three reported missense mutations at N582 cause moderate to severe hemophilia A (N582D, N582H, N582K). These mutations are termed glycosylation defective mutations in the FVIII variant database (Table 1). We introduced these three missense mutations into the A2 domain expression construct and analyzed the expression of mutant constructs 36 hours after transfection. We compared the secretion levels of these mutants to those of WT A2 domain in conditioned media. Cells were fed with fresh media and conditioned media were collected at 0.5, 1, 2 and 4 hours. While increasing amounts of the WT A2 were detected in conditioned media over time, all three A2 mutants were undetectable (Fig. 1C). There were no differences in intracellular protein levels between WT A2 and these A2 mutants in COS-1 cells (Fig. 1C). We also investigated WT and N582 mutant A2 secretion in cells incubated at 28 °C for 24 h. While WT A2 was readily detected in conditioned media at 2 and 4 h of chase, no mutant A2 domains were detected (Fig. 1D). Therefore, lowering the temperature, which may stabilize misfolded proteins or increase the extent of proper protein folding in cells^37^, is not sufficient to increase the secretion of these three mutants. Again, there were no obvious differences in intracellular protein levels between WT and mutant forms of the A2 domain (Fig. 1D).

### Certain missense mutations near N582 lead to abnormal N-glycosylation of the A2 domain

**Figure 2:**
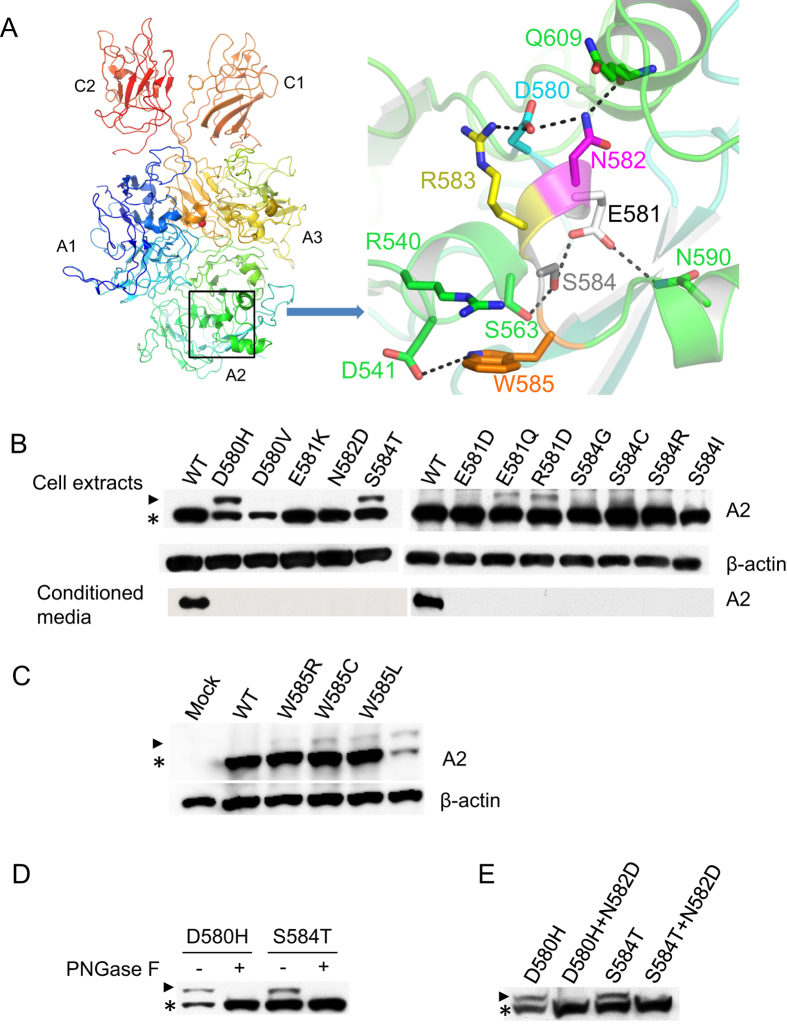
Glycosylation status of the A2 domain with missense mutations adjacent to N582 (D580 to W585). (**A**) The amino acid residues of D580 to S584 form a short 3_10_-helix in the structure of B domain deleted FVIII heterodimer. The right side of the panel depicts a hydrogen-bonding network mediated by the sidechains of the residues that comprise the 3_10_ helix. W585 is also highlighted on the right panel. Residues and secondary structure elements outside the helix are colored green. The FVIII structure is based on entry 2R7E in the Protein DataBank^35^. (**B**) Extracts of COS-1 cells transiently transfected with constructs expressing WT A2 and the indicated A2 mutants were collected at 36 h after transfection and analyzed by 10% SDS-PAGE and immunoblotting. Arrowhead indicates glycosylated A2 domain and asterisk indicates non-glycosylated A2 domain. Protein levels in the media were detected by immunoprecipitation with an anti-Flag antibody followed by immunoblotting. (**C**) Extracts of COS-1 cells transiently transfected with constructs expressing WT A2 and the indicated A2 mutants were collected at 36 h after transfection and analyzed by 10% SDS-PAGE and immunoblotting. (**D**) Cell extracts of D580H and S584T mutants were treated with PNGase F and analyzed by immunoblotting. (**E**) The N582D mutation was introduced to the D580H and S584T mutants. Cell extracts expressing single or double mutants were analyzed by immunoblotting. Arrowhead denotes N-glycosylated forms and asterisk denotes non-glycosylated form of the A2 domain. All experiments were carried out independently 2–3 times.

The N582 residue is located in the middle of a 3_10_-helical turn or short helix composed of residues D580 to S584 (Fig. 2A). Hemophilia A mutations have been reported for all 5 amino acids in this helix. In fact, all of these residues other than R583 exhibit multiple mutations (Table 1). We introduced all 11 hemophilia A mutants in this helix into the A2 domain construct and transiently transfected the mutant constructs into COS-1 and HEK293T cells. Except for the D580V mutant, which resulted in an extremely low intracellular protein level, all other mutants were expressed at levels similar to that of the WT A2 domain (Fig. 2B and Figure S2B). Of these mutants, two bands were observed for D580H, E581D, E581Q, R583G and S584T in cell extracts. In each case, the lower band exhibited mobility similar to the WT A2 domain. The upper band of E581D was extremely weak, but detectable. We also noted that a residue adjacent to the 3_10_-helical turn (W585) is also associated with multiple mutations. All 3 amino acid mutations (W585R, W585L and W585C) were introduced into the A2 expression construct and analyzed for their glycosylation status in COS-1 and HEK293T cells (Fig. 2C and Figure S2B). All three A2 mutants produced a minor upper band with the same mobility as the upper band of the D580H mutant (Figure S2B). We selected the D580H and S584T mutants to determine whether the upper bands were due to abnormal N-linked glycosylation of the A2 domain. The upper bands disappeared and only the lower band was detected in SDS-PAGE after PNGase F treatment (Fig. 2D). To further demonstrate that the N582 residue was glycosylated, we introduced a second mutation, N582Q, to the D580H and S584T mutants. Both double mutants produced only the lower band in SDS-PAGE (Fig. 2E). These results indicate that neighboring residues influence the availability of N582 for glycosylation. All four mutations that resulted in aberrant N582 glycosylation also severely reduced the secretion of the A2 domain, as no mutant proteins were detected in conditioned media (Fig. 2B).

**Figure 3:**
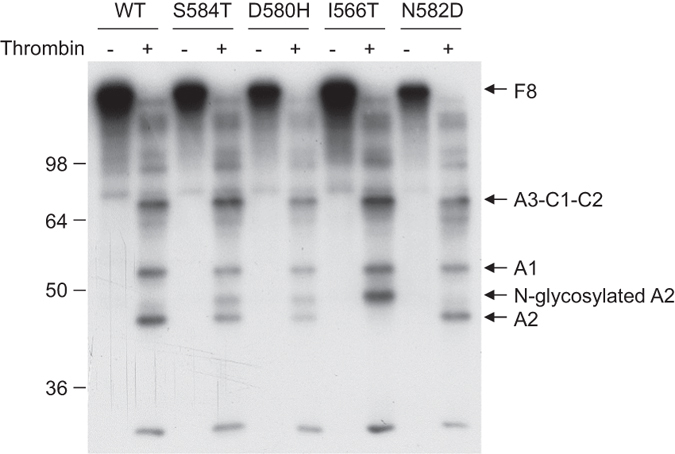
Thrombin cleavage products of metabolically labeled FVIII. COS-1 cells were transfected with pTM2-FVIII expressing WT or S584T, D580H, I566T and N582D mutants and labeled with [35S]-methionine/cysteine. FVIII was immunoprecipitated with anti-FVIII beads and digested with thrombin or left untreated. Samples were separated by reducing SDS-PAGE. Indicated are FVIII and specific thrombin cleavage products of FVIII. Representative images of two independent experiments are shown.

To determine whether the partial glycosylation of A2 domain mutants also occurs in the context of full-length FVIII, we introduced the S584T, D580H and N582D mutations into the full-length FVIII expression construct pTM2-FVIII. In addition, we also introduced the I566T mutation, which introduces a new N-glycosylation site that was previously reported to be glycosylated^29^. WT and mutant FVIII constructs were transfected into COS-1 cells and metabolically labeled with ^35^S, immunoprecipitated with conjugated anti-FVIII beads, and digested with thrombin. Thrombin cleavage products were separated by SDS-PAGE and visualized by radiography. The A2 domain of WT and N582D FVIII produced a single band of higher mobility, while the A2 domain of I566T FVIII produced a single band with decreased mobility. Both S584T and D580H mutants produced A2 domain doublets that correspond to the faster-migrating band from I566T and the slower-migrating band from WT and N582D FVIII (Fig. 3). These results indicate that mutations that lead to glycosylation of isolated A2 domain also lead to glycosylation of the A2 domain in full-length FVIII.

### Protease susceptibility of WT and mutant A2 domain of FVIII

**Figure 4:**
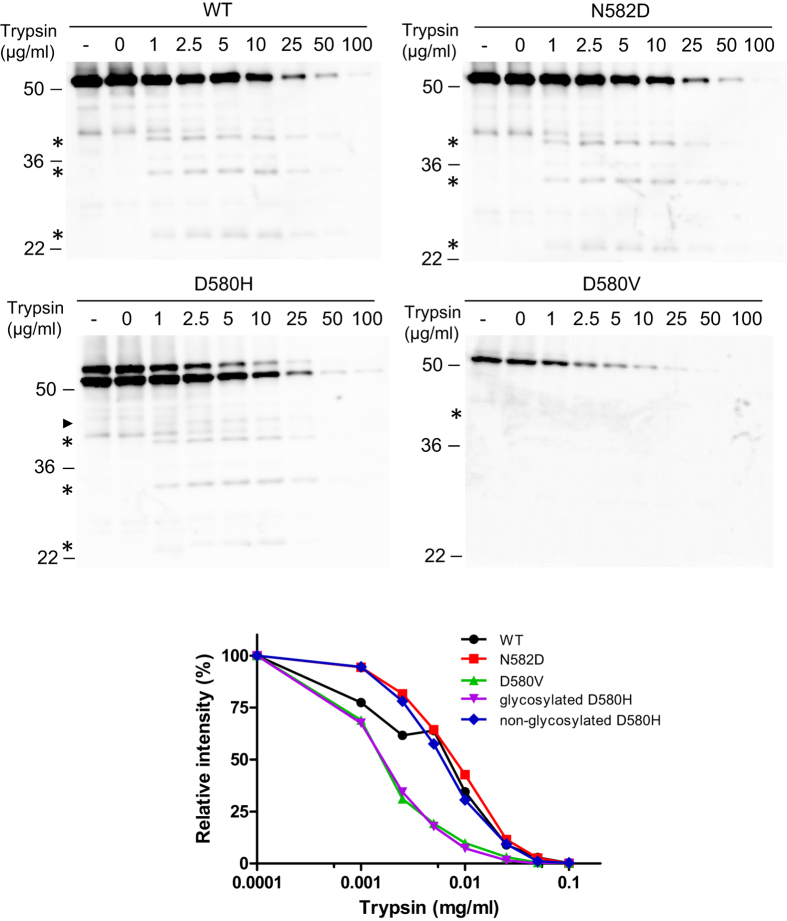
Protease susceptibility of WT and mutant FVIII A2 domain. Lysates from COS-1 cells transfected with the WT A2, A2-N582D, A2-D580H or A2-D580V plasmids were subjected to limited proteolysis at the indicated trypsin concentrations. Samples were immunoblotted with a rabbit anti-Flag antibody. The first lane of each panel contains an undigested sample prior to incubation on ice. Asterisks indicate partially digested intermediates. Arrowhead in the D580H panel denotes the digestion intermediate with N-linked glycan. Densities of remaining undigested A2 domain were quantified and plotted as percentages of levels of control samples incubated on ice without added trypsin (second lane).

Missense mutations may cause misfolding of the A2 domain and render it more susceptible to limited proteolytic digestion^38^,^39^. We digested the WT, D580H, D580V and N582D mutants of A2 with 0–100 μg/ml of trypsin on ice for 15 minutes to examine protease resistance. The primary band disappeared with increasing amounts of trypsin. Several partially digested bands were also detected at lower trypsin concentrations, including one band that contains the glycosylation sequon (Fig. 4). We found the degradation rates of WT, N582D and the non-glycosylated D580H mutant to be similar. Each exhibits 50% digestion at trypsin concentrations between 5–10 μg/ml. The N-glycosylated D580H species and the D580V mutant, however, were more sensitive to trypsin with a 50% trypsin digestion concentration between 1–2.5 μg/ml, suggesting that glycosylation of the D580H mutant and the D580V mutation result in a more labile or less stable A2 domain. Similar results were obtained when the reactions were carried out at 37 °C (Figure S3).

### Proteasome mediates the degradation of abnormally glycosylated A2 and A2 D580V

**Figure 5:**
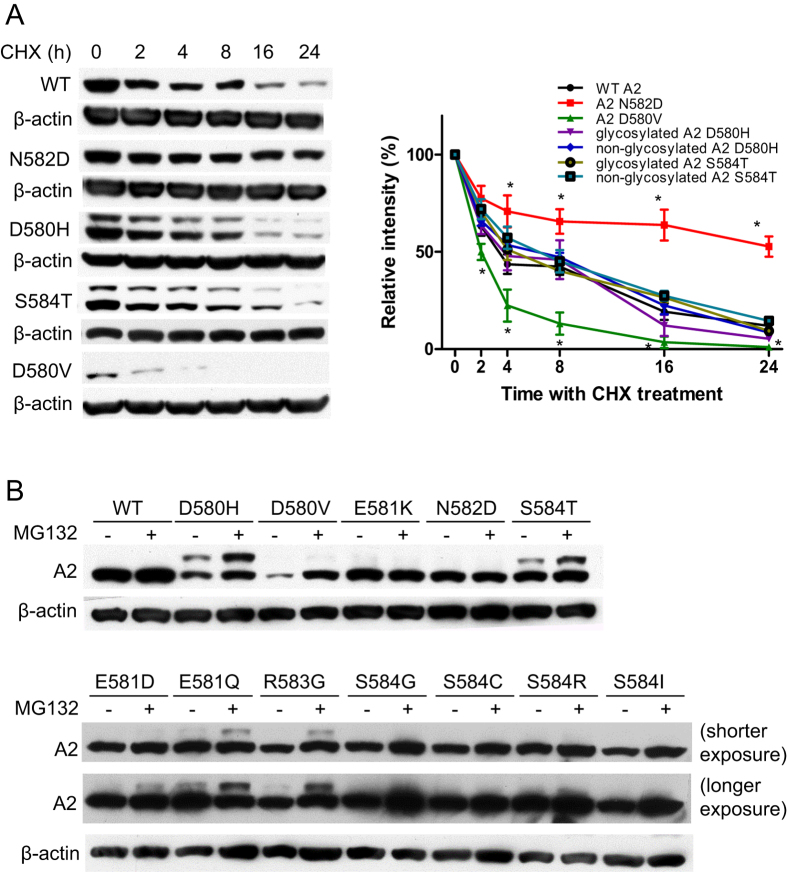
Stability and proteasome degradation of A2 mutants. (**A**) Twenty-four hours after transfection with the indicated expression constructs, HEK293T cells were treated with 200 μM cycloheximide (CHX) for 30 m and chased for 24 h. Cell extracts were harvested at 0, 2, 4, 8, 16 and 24 h and analyzed by immunoblotting with anti-Flag or anti-β-actin antibody. Relative protein levels during the CHX chase were quantified and plotted as fraction remaining (data are mean ± SEM, n = 3. *P < 0.05). (**B**) Twenty-four hours after transfection with the indicated expression constructs, HEK293T cells were treated with DMSO (−) or 10 μM MG132 (+) for 12 hours. Cell extracts were analyzed by immunoblotting with anti-Flag or anti-β-actin antibodies. Two different exposures were taken for the lower panel of the anti-Flag blots to show differences between DMSO and MG132 treatments. All experiments were performed independently three times.

Next, we used cycloheximide (CHX) to block protein translation and carried out a CHX chase to compare the intracellular decay rates of four mutants, D580H, D580V, N582D and S584T in HEK293T cells. The WT A2 domain had a half-life of ~5.6 h with relatively slower degradation kinetics during the first 8 h after cycloheximide treatment (Fig. 5A). At 24 h, approximately 12% of the A2 domain remained inside cells. In contrast, the decay rate of the N582D mutant was much slower with a half-life of 27.9 h, and about 50% remained inside cells after 24 hours. The decay rate and pattern of glycosylated or non-glycosylated A2 D580H were similar to those of WT A2. The half-life of the non-glycosylated D580H mutant was about 6.3 h and the half-life of the glycosylated D580H mutant was just slightly shorter, at 5.4 h. Since the majority of the D580H mutant was not secreted, the degradation rate of the D580H mutant was comparable to the secretion rate of WT A2. Correlating with its susceptibility to trypsin digestion, the decay of the D580V mutant was more rapid, with a half-life of only 2.1 hours, suggesting that the mutation generates a very unstable protein that is rapidly degraded.

As part of ERAD, proteins that fail ER quality-control checkpoints are dislocated into the cytoplasm and degraded by the 26S proteasome. We used the proteasome inhibitor MG132 to explore the degradation mechanism of A2 missense mutations. MG132 was added to cell culture media 24 hours after transfection. After 12 h of treatment with 10 μM MG132, the intracellular level of the D580V mutant was increased approximately 4-fold (Fig. 5B). Although the total protein levels of D580H and S584T mutants increased modestly, the glycosylated forms of these mutants were elevated approximately 3-fold (Fig. 5B). On the other hand, protein levels of the E581K and N582D mutants were not affected by MG132. These results reveal varying degrees of proteasome-dependent degradation between A2 mutants. They also suggest that the lack of secretion of selected mutant A2 domains is not necessarily due to rapid degradation or clearance from the ER during their biogenesis. In general, however, the glycosylated forms are more sensitive to MG132 inhibition, suggesting that aberrant glycosylation is a particularly strong trigger for dislocation from the ER and proteasome-mediated degradation.

### Increased interactions between A2 missense mutants and ER chaperones

**Figure 6:**
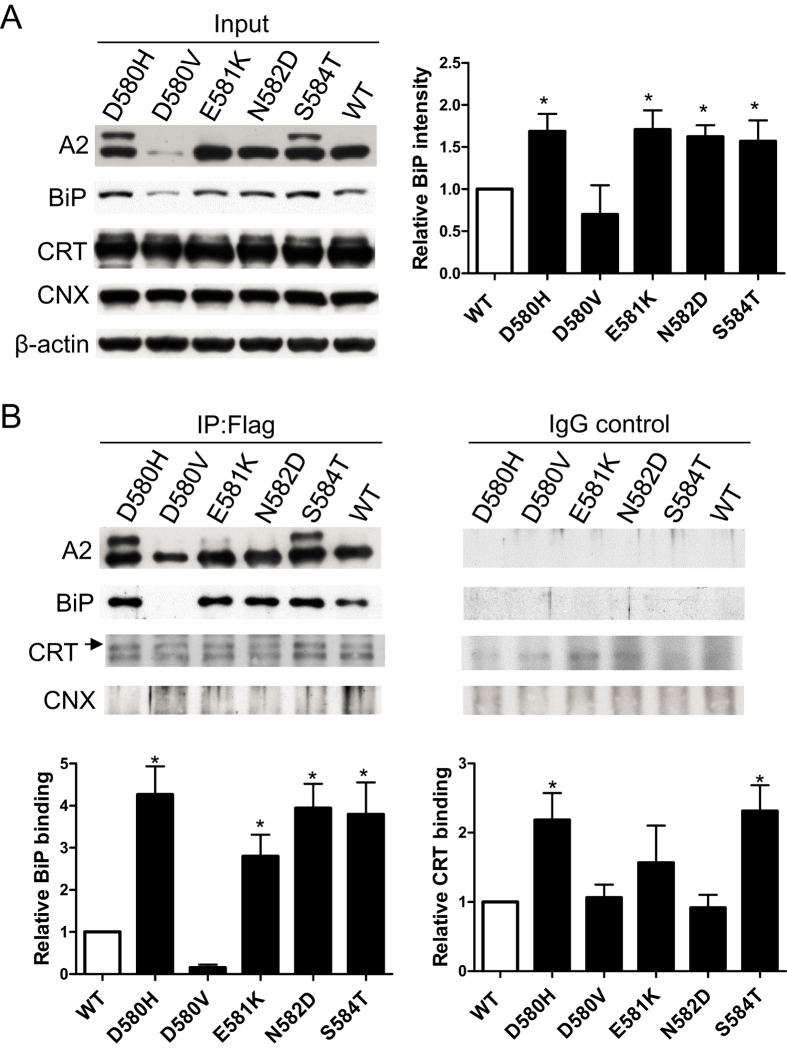
Inductions of ER chaperones and increased interaction with BiP by selected A2 mutants. (**A**) Thirty-six hours after transfection, cell extracts were analyzed by immunoblotting with the indicated antibodies. CRT: calreticulin, CNX: calnexin. BiP intensities were densitometrically quantified and expressed as fold changes compared to WT after normalizing to the β-actin loading control (data are mean ± SEM, n = 2. *P < 0.05). (**B**) Thirty-six hours after transfection, cell extracts were immunoprecipitated with mouse monoclonal anti-Flag antibody or normal mouse IgG. The immunoprecipitates were analyzed by immunoblotting with the indicated antibodies. The amounts of BiP and CRT that co-immunoprecipitated with A2 domain mutants were densitometrically quantified and expressed as fold changes relative to WT (data are mean ± SEM, n = 3. *P < 0.05).

We investigated the interactions between ER chaperones and five representative A2 mutants, D580H, D580V, E581K, N582D and S584T. Thirty-six hours after transfection with plasmids of WT A2 and A2 mutants, we collected cell extracts to analyze protein levels of BiP, CRT and CNX. Expression of all mutants other than D580V increased the protein levels of BiP to 1.5- to 2-fold of the WT A2 (Fig. 6A). However, there were no differences in protein levels of CRT or CNX between WT and mutant A2 in cell lysates (Fig. 6A). Next, we carried out immunoprecipitations (IPs) to detect the interactions of ER chaperones with the A2 mutants. With the exception of D580V, A2 mutants all associated with BiP to a greater extent compared to WT A2. The amounts of BiP that co-immunoprecipitated with these mutants increased 1.6- to 2.8-fold over WT A2 (Fig. 6B). Interactions of the A2 mutants with BiP appear to be inversely correlated to their secretion levels. None of the mutants induced expression of CRT or CNX. However, binding of CRT increased with D580H and S584T mutants, which are partially glycosylated (Fig. 6B). No obvious interactions of WT A2 or A2 mutants with CNX were detected.

### Antigen and activity levels of full-length FVIII mutants

**Figure 7:**
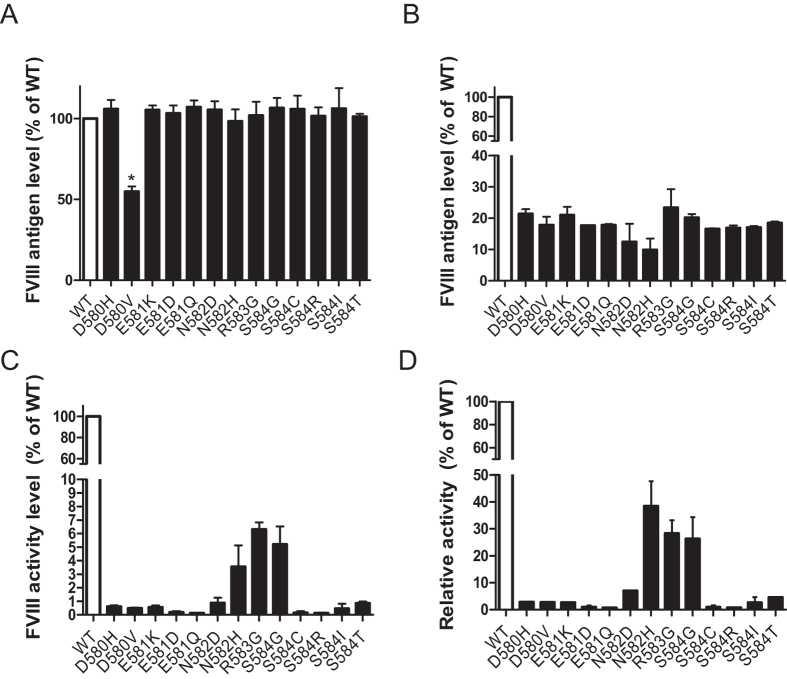
Antigen and activity levels of full-length FVIII mutants. (**A**) and (**B**) Forty-eight hours after transfection, FVIII antigen levels in cell extracts (**A**) and conditioned media (**B**) were analyzed by ELISA. Antigen levels of FVIII mutants were plotted as percentages of WT FVIII (data are mean ± SEM, n = 3). Only the level of D580V mutant was significantly reduced comparing to WT FVIII (*P < 0.05). (**C**) Forty-eight hours after transfection, activity levels of FVIII in conditioned media were analyzed by a chromogenic assay. The averages of mutants were plotted as percentages of WT FVIII activity (n = 3). Activities of all the mutants were significantly reduced compared to WT FVIII (*P* < 0.05). (**D**) Relative activity for a given mutant was calculated as the ratio of the activity level and the antigen level in conditioned media, plotted as percentages of the WT FVIII relative activity. Relative activities of all mutants were significantly reduced compared to WT FVIII (*P* < 0.05).

We introduced the missense mutations in the 3_10_-helix described above into a full-length FVIII expression plasmid (pMT2-FVIII). Forty-eight hours after transfection, cell extracts and conditioned media of COS-1 cells were harvested to detect the antigen level or activity level of FVIII. The protein level of FVIII D580V inside cells was lower than that of WT FVIII (Fig. 7A), consistent with the A2 domain results (Fig. 2). There were no significant differences in protein levels between the other mutants and WT FVIII in cell extracts. However, antigen levels of all the FVIII mutants in conditioned media were less than 30% of the WT FVIII level (Fig. 7B). Therefore, all the mutants likely cause cross-reacting material (CRM) reduced hemophilia A which is categorized on the basis of decreased secretion of FVIII to below 30% of the WT FVIII level.

We used a chromogenic assay to measure the activity of FVIII mutants in conditioned media secreted from transiently transfected COS-1 cells. The activities of most mutants were less than 1% of that of WT FVIII (Fig. 7C), consistent with the mutant phenotypes in hemophilia patients (Table 1). Severity of the S584I mutant was not reported and our results suggest that it is a severe mutation. The activities of N582H, R583G and S584G FVIII mutants were between 3–7% in our study, consistent with the mild to moderate phenotype of hemophilia A in patients carrying these mutations. By normalizing the activities by the corresponding antigen levels, we determined a relative activity for each mutant secreted into conditioned media. Except for N582H, R583G and S584G mutations, the relative activities of all other FVIII mutants were less than 5% of that of WT FVIII (Fig. 7D). These data suggest that perturbations of the A2 domain induced by mutations near N582 affect both the secretion and the overall conformation and stability of full-length FVIII, as gauged by differential effects on secretion and enzymatic activity.

## Discussion

Our results confirm that all of the consensus sequences outside the B domain are glycosylated in cells, with the notable exception of the A2 domain consensus site residue N582. Expression of isolated FVIII domains allowed detection of partially glycosylated species, such as the minor non-glycosylated A3 domain. A recent quantitative proteomic study found that N1810 at the A3 domain is ~80% occupied by an N-linked glycan (American Society of Hematology 58^th^ Annual Meeting abstract #3765), consistent with our observation. Previous studies have compared N-glycosylation of recombinant FVIII expressed in different cell types, as well as plasma-derived FVIII (synthesized in endothelial cells *in vivo*). Full-length FVIII is N-glycosylated in 21 of 25 potential sites. Among the four N-glycans outside the B domain, N41 and N1810 carry complex structure, while N239 and N2118 are of mostly high-mannose type^34^,^36^. No differences in utilization of these glycosylation sites were noted among plasma-derived and recombinant FVIII expressed in different cell types^33^,^34^. Differences in N-glycosylation patterns are mainly due to differences in structures of N-glycans^34^,^40^,^41^, which are determined by Golgi-localized modification enzymes. In our study, we expressed recombinant proteins in both COS-1 cells and HEK293T cells and obtained the same N-glycosylation patterns for all mutants. N-glycosylation is mainly a co-translational event^10^. Nevertheless, one concern is whether N-glycosylation observed in isolated A2 domain is relevant in the context of the full-length FVIII. Our results show identical N-glycosylation patterns between isolated A2 domain and full-length FVIII (Fig. 3).

A number of factors may influence whether N-glycosylation consensus sites are utilized or skipped. In general, N-X-T sequons are better substrates for OST than N-X-S sequons, potentially explaining the partial glycosylation of the S584T mutant. The middle residue and the flanking sequences may also affect glycosylation efficiency^42^,^43^,^44^,^45^,^46^,^47^. In the case of FVIII however, mutations that lead to partial glycosylation of N582 (D580H, E581Q, R583G) either do not change native residues to those correlated with increased glycosylation, or are associated with only a slight increase in glycosylation. The W585 residue that follows the NRS sequon (the Y position) in the A2 domain is an amino acid that may inhibit glycosylation of N582 based strictly on sequence analyses^48^. However, all three hemophilia mutations of this residue result in relatively limited glycosylation of the A2 domain (Fig. 2C), suggesting that W585 has only a minor inhibitory effect on the glycosylation of N582. Statistical studies suggest that local conformation can influence the efficiency of glycosylation^49^. Recent structural studies show that the sidechains of the target N and S/T residues of the sequon interact with OST active site groups, and the target asparagine amide is directly activated by the enzyme for oligosaccharyl transfer^50^,^51^,^52^. In contrast, residues 580–584 engage in a network of hydrogen bonds that stabilize the local 3_10_-helical configuration (Fig. 2A). D580 is engaged in a hydrogen bond network with the sidechains of N582 and R583 that stabilizes the 3_10_-helix. At the other end of the helix, the S584 sidechain is involved in a hydrogen-bonding network with E581 and sidechains from several nearby α-helices (Fig. 2A). We speculate that this short but highly stabilized 3_10_-helix folds too rapidly for the OST to transfer glycan to the amine group of N582. Mutations could destabilize the 3_10_-helix or delay the folding of this secondary structure so that N582 becomes partially accessible to OST^53^.

Although all mutations in the 3_10_-helix found in hemophilia A patients resulted in defective secretion of the A2 domain, these mutations lead to different intracellular trafficking defects. Notably, the D580V mutation leads to markedly decreased intracellular protein level, due to increased proteasomal degradation. It is noteworthy that the D580 sidechain is loosely surrounded by hydrophobic sidechains, including A544, V578, I613, and A644. This loose hydrophobic cavity increases the strength of hydrogen bonds and salt bridges. The D580V mutation could generate an extremely perturbed A2 domain, potentially by leading to collapse of the 580–584 sequence into the protein. Such a perturbed domain, either by itself or in the context of FVIII, would be highly susceptible to ERAD. The glycosylated A2-D580H was more sensitive to trypsin than WT A2 and the non-glycosylated A2-D580H, suggesting that the addition of a bulky N-glycan further disrupts the conformation. Compared to D580V, N582D has milder effects on the stability of the A2 domain. The A2-N582D mutant was degraded slowly inside cells and its sensitivity to trypsin digestion was similar to that of WT-A2, in keeping with the ability of aspartic acid to recapitulate the hydrogen-bonding network of asparagine. However, the proximity of two negatively charged residues in this mutant (D580 and D582) may nevertheless reduce the stability of the local structure and perturb overall A2 domain conformation, as the N582D mutant is not secreted (Fig. 1C,D) and associates more strongly with chaperones than does the WT-A2 domain (Fig. 6). That the conservative substitution S584T leads to a severe secretion defect is somewhat surprising. However, the substitution of S by T in this densely packed region may disrupt the local hydrogen bonding network by clashing or repelling the sidechain of E581, knocking the latter out of position. Therefore, S584 mutations may have similar effects as E581 mutations.

The decay rate of WT A2 inside cells is determined by both secretion and degradation. The relatively low secretion rates of A2 mutants imply that their degradation rates are the main determinants of their overall decay rates. The decay rates of glycosylated or non-glycosylated A2-D580H and A2-S584T were similar to that of WT A2. Therefore, the degradation rate of glycosylated or non-glycosylated A2-D580H and A2-S584T inside cells were comparable to the secretion rate of WT A2 domain. Degradation of all glycosylated form of A2 mutants was blocked by MG132, suggesting that the glycosylated A2 mutants undergo degradation mainly through the proteasome pathway. However, different non-glycosylated mutants react differently to MG132 treatment. While the D580V mutant appears most actively degraded by the proteasome, E581K and N582D mutants are insensitive to MG132 (Fig. 5B), suggesting a proteasome-independent pathway for the degradation of these mutants. Thus, mutations in the 3_10_-helix have different effects on the stability of the A2 domain.

Chaperones play essential roles in protein folding and quality control of FVIII^23^. BiP interacts with all the misfolded A2 mutants, whereas CRT is more specific for A2 mutants with abnormal N-linked glycosylation, suggesting that these mutants robustly engage the CRT cycle. The correlation between aberrant N-linked glycosylation, CRT-binding and preferential stabilization of the glycosylated species by MG132 suggests that the CRT cycle effectively diverts aberrantly glycosylated A2 domains to the ERAD machinery for dislocation and proteasomal degradation. The D580V mutant may not extensively induce BiP and CRT due to its low stability and rapid degradation (Figs 4 and 5). However, it is also highly stabilized upon MG132 treatment (Fig. 5B), suggesting that it is processed by other elements of the ER lumenal quality control machinery to ensure its degradation.

It is intriguing that there is a relatively limited range of secretion for all full-length FVIII mutants, falling approximately between 10–20% of WT FVIII levels (Fig. 7B). Full-length FVIII secretion is higher than that of individual A2 domain mutants, which are undetectable in conditioned media (Figs 1 and 2). Given that these mutants all have in common a defective or perturbed A2 domain, it appears that the integrity of the other domains is sufficient to ensure proper trafficking and secretion at these base levels, but no more. The activity levels of secreted mutants are further reduced to levels that are consistent with the phenotypes of the corresponding patients (Fig. 7C,D). Amino acid residues 580–584 are not located in the known functional regions of the A2 domain that mediate FIX activation and thrombin activation^1^. Decreased activities suggest that in addition to affecting secretion, these mutants also have negative effects on FVIII function that result from disruption of the conformation of the A2 domain. Concomitantly, the overall conformation of FVIII may be disturbed as well. While secretion of these mutants was decreased, protein levels within cells were not significantly elevated (Fig. 7A), suggesting that proteostatic control prevents excessive accumulation of misfolded proteins.

In conclusion, our results demonstrate that the N-glycosylation sequon in the A2 domain is located in a structural element that is critically required for proper folding and conformation of FVIII. Local structural stability of this 3_10_-helix may be the reason why this sequon is skipped by OST. Although all mutations in this region lead to secretion-defective and function-compromised FVIII, the specific defects on protein conformation and intracellular trafficking vary among different mutations.

## Methods

### Plasmid construction

Individual domains of wild-type (WT) FVIII were cloned into the pED (A1 (1–379) and A2 (380–711)) or pZ (A3 (1689–2019) and C (2020–2332)) expression vectors^19^. All constructs were engineered to lead with a signal sequence from calreticulin, followed by a Flag tag sequence, a linker sequence and a FVIII domain. Missense mutations were introduced into the pED-Flag-A2 and the pMT2-FVIII^33^ plasmids using the QuickChange site-directed mutagenesis II XL kit (Agilent) and confirmed by Sanger sequencing. Primer sequences used for mutagenesis are listed in Table S1. For full-length FVIII mutagenesis, we used the restriction enzymes KpnI and EcoRV to remove a DNA fragment containing confirmed mutant A2 domain from the corresponding mutagenized pMT2-FVIII construct, and ligated it back into KpnI/EcorRV-digested WT pMT2-FVIII. The resulting constructs were re-sequenced to confirm the presence of mutations. Nucleotide numbering of FVIII gene mutations is based on HGVS recommendations, so that the A of the ATG-translation initiation codon corresponds to nucleotide 1. The first amino acid after signal peptide cleavage corresponds to amino acid residue 1.

### Reagents

Monoclonal antibodies against Flag, CRT and CNX were purchased from Sigma (St. Louis, MO) and the monoclonal anti-BiP was from Santa Cruz Biotechnology (Santa Cruz, CA). Monoclonal anti-human FVIII antibody, a gift from D. Pittman (Bayer), was conjugated to the AminoLink coupling gel agarose beads ThermoFisher (Waltham, MA). Cycloheximide (CHX) and MG132 were obtained from Sigma and the Peptide-N-Glycosidase F (PNGase F) enzyme was purchased from the New England BioLabs (Ipswich, MA). Protein A/G Plus-agarose beads were purchased from Santa Cruz Biotechnology (Santa Cruz, CA).

### Cell culture

HEK293T and COS-1 cells were grown in DMEM supplemented with 10% FBS, 100 IU/ml penicillin and 100 IU/ml streptomycin at 37 °C and in 5% CO_2_. Cells were transfected using FuGENE 6 (Roche, Indianapolis, IN) according to the manufacturer’s instruction. Cells and conditioned media were harvested at various times after transfection for analysis. To control for variation in transfection efficiencies, experiments were independently performed 2–3 times. For stability assays, cells were treated with 200 μM CHX or 10 μM MG132 at 24 h post transfection. For CHX chase assays, cells were chased for 0, 2, 4, 8, 16, 24 hours, harvested, and lysed. After 12 h treatment with MG132, cell extracts were harvested for Western blot analysis.

### Trypsin digestion

Cells were lysed in NP-40 lysis buffer (50 mM Tris-HCl, pH 7.5, 150 mM NaCl, 1% Nonidet P-40, 0.05% SDS, 2 mM CaCl_2_) 36 h after transfection with WT A2, A2-N582D, A2-D580H or A2-D580V plasmids. After removal of debris by centrifugation (15,000 *g*, 10 min, 4 °C), 30 μg proteins were digested on ice for 15 min by varying concentrations of trypsin (0–100 μg/ml) in 15 μl reaction volume.

### Western blotting and immunoprecipitation

Cells were lysed in NP-40 lysis buffer supplemented with EDTA-free protease inhibitor cocktail (Roche Applied Science) on ice for 10 minutes. Lysates were cleared by centrifugation (15,000 *g*, 10 min, 4 °C). Aliquots of cell lysates were separated by 10% SDS-polyacrylamide gel electrophoresis (PAGE), and Western blot analysis was performed using the appropriate antibodies. Images of chemiluminescent blot signals from horseradish peroxide-conjugated secondary antibodies were scanned and analyzed using ImageJ software^34^. Band intensities were quantified using Gel Analysis following the software’s user guide.

For immunoprecipitation, cell lysates or media were precleared with 20 μl of protein A/G Plus-agarose beads to remove nonspecific binding proteins. Precleared cell lysates were incubated with mouse anti-Flag antibody or normal mouse IgG overnight at 4 °C and then incubated with 20 μl of protein A/G Plus-agarose beads (Santa Cruz, CA) for 1 hour at 4 °C. Beads were collected by centrifugation at 7,000 rpm for 1 min, washed three times with the NP-40 buffer and incubated with 40 μl SDS sample buffer at 100 °C 5 minutes. The supernatant was harvested and separated in a 10% SDS-PAGE gel. Western blot analysis was performed using appropriate primary antibodies.

### Metabolic labelling and thrombin digestion of FVIII

Twenty hours after transfection, COS-1 cells transfected with PMT2-FVIII plasmids were metabolically labeled with [35S]-methionine/cysteine (250 μCi/mL in methionine/cysteine-free DMEM) (MP Biomedicals) for 45 minutes, followed by a 30-minute incubation in complete medium. Cell extracts were prepared by lysis in the NP-40 lysis buffer and immunoprecipitated with anti-FVIII antibody coupled to CL-4B sepharose. Immunoprecipitated proteins were washed with NP-40 lysis buffer and PBS, and resuspended in 50 mM Tris-Hcl pH 7.5, 150 mM NaCl, 2.5 mM CaCl_2_ and 5% glycerol (buffer A). Immunoprecipitated FVIII in buffer A were divided into two aliquots for incubation in the absence or presence of 5 U/ml thrombin (Sigma) at 37 °C for 30 minutes. The resulting samples were separated in a 12% SDS-PAGE gel and visualized by exposing to an X-ray film using a Kodak Biomax Transcreen LE.

### FVIII activity and antigen detection

Forty-eight hours after transfection, conditioned media were collected for FVIII activity and antigen assays. The Coatest SP4 Factor VIII kit (Chromogenix, Bedford, MA) was used to measure FVIII activity in conditioned media. WT mouse plasma was used as standards for activity assays. The VisuLize FVIII antigen kit (Affinity Biologicals, Ancaster, Canada) was used to analyze the antigen levels of FVIII in cell lysates and conditioned media. Activities and antigen levels of WT and mutant FVIII were subtracted from mock-controls.

### Statistical analysis

All data are presented as mean ± SEM. Statistical significance was calculated using two-tailed Student’s t test. p-values < 0.05 were considered significant for all assays.

## Additional Information

**How to cite this article:** Wei, W. *et al*. Missense mutations near the N-glycosylation site of the A2 domain lead to various intracellular trafficking defects in coagulation factor VIII. *Sci. Rep.* **7**, 45033; doi: 10.1038/srep45033 (2017).

**Publisher's note:** Springer Nature remains neutral with regard to jurisdictional claims in published maps and institutional affiliations.

## Supplementary Material

Supplementary Information

## Figures and Tables

**Table 1:** Missense mutations found in the 3_10_-helix formed by D580-S584 in hemophilia A patients.

Mutation	Mutation Effect	Reported Severity
c.1795G > C	p.D580H	Severe
c.1796A > T	p.D580V	Severe
c.1798G > A	p.E581K	Severe
c.1798G > C	p.E581Q	Severe
c.1800G > T	p.E581D	Severe
c.1801A > C	p.N582H	Moderate
c.1801A > G	p.N582D	Severe
c.1803C > G	p.N582K	Severe
c.1804C > G	p.R583G	Mild
c.1807A > G	p.S584G	Mild
c.1807A > T	p.S584C	Severe
c.1808G > C	p.S584T	Severe
c.1808G > T	p.S584I	Not reported
c.1809C > G	p.S584R	Moderate/Severe
c.1810T > C	p.W585R	Severe
c.1811G > T	p.W585L	Moderate
c.1812G > C	p.W585C	Severe
c.1812G > T	p.W585C	Severe
