# Supplementary material for: Missense mutations near the N-glycosylation site of the A2 domain lead to various intracellular trafficking defects in coagulation factor VIII
Source: Sci Rep. 2017 Mar 22;7:45033. doi: 10.1038/srep45033 (PMC5361195; doi:10.1038/srep45033)
Supplement: Supplementary Information [file srep45033-s1.pdf]

Missense mutations near the N-glycosylation site of the A2 domain lead to various intracellular trafficking defects in coagulation factor VIII

Wei Wei[1,2], Chunlei Zheng[1], Min Zhu[3], Xiaofan Zhu[2], Renchi Yang[2], Saurav Misra[4], Bin Zhang[1]

[1]Genomic Medicine Institute, Cleveland Clinic Lerner Research Institute, Cleveland, OH

[2]State Key Laboratory of Experimental Hematology, Institute of Hematology and Blood Diseases Hospital, Chinese Academy of Medical Sciences & Peking Union Medical College, Tianjin, China

[3]Department of Pathology, Karamay Central Hospital, Karamay, Xinjiang, China

[4]Department of Biochemistry and Molecular Biophysics, Kansas State University, Manhattan, KS

¶Corresponding authors:

Genomic Medicine Institute
Cleveland Clinic Lerner Research Institute
9500 Euclid Ave/NE50
Cleveland, OH 44195
Email: zhangb@ccf.org
Phone: 216-444-0884
Fax: 216-636-0009

Running title: Missense mutations in A2 domain of FVIII

Table S1. Mutagenesis primers used in the study

**Mutation (codon change)**		**Primer sequence**
D580H (GAT>CAT)	Sense	cagcttcggttctcatgaaatacagaaaacaggatgacattcc
	Antisense	ggaatgtcatcctgttttctgtatttcatgagaaccgaagctg
D580V (GAT>GTT)	Sense	taccagcttcggttctcaacaaatacagaaaacaggatg
	Antisense	catcctgttttctgtatttgttgagaaccgaagctggta
E581K (GAG>AAG)	Sense	ccagcttcggttcttatcaaatacagaaaacaggatgaca
	Antisense	tgtcatcctgttttctgtatttgataagaaccgaagctgg
E581Q (GAG>CAG)	Sense	ccagcttcggttctgatcaaatacagaaaacaggatgaca
	Antisense	tgtcatcctgttttctgtatttgatcagaaccgaagctgg
E581D (GAG>GAT)	Sense	aggtaccagcttcggttatcatcaaatacagaaaacagg
	Antisense	cctgttttctgtatttgatgataaccgaagctggtacct
N582H (AAC>CAC)	Sense	tgaggtaccagcttcggtgctcatcaaatacagaaaac
	Antisense	gttttctgtatttgatgagcaccgaagctggtacctca
N582D (AAC>GAC)	Sense	tgaggtaccagcttcggtcctcatcaaatacagaaaac
	Antisense	gttttctgtatttgatgaggaccgaagctggtacctca
N582K (AAC>AAG)	Sense	gaggtaccagcttcgcttctcatcaaatacagaaaac
	Antisense	gttttctgtatttgatgagaagcgaagctggtacctc
R583G (CGA>GGA)	Sense	tgtgaggtaccagcttccgttctcatcaaatacag
	Antisense	ctgtatttgatgagaacggaagctggtacctcaca
S584R (AGC>GGC)	Sense	ttctctgtgaggtaccaccttcggttctcatcaaa
	Antisense	tttgatgagaaccgaaggtggtacctcacagagaa
S584C (AGC>TGC)	Sense	gtgaggtaccagcatcggttctcatcaaatacagaaaa
	Antisense	ttttctgtatttgatgagaaccgatgctggtacctcac
S584T (AGC>ACC)	Sense	tctgtgaggtaccaggttcggttctcatcaaatac
	Antisense	gtatttgatgagaaccgaacctggtacctcacaga
S584I (AGC>ATC)	Sense	ctctgtgaggtaccagattcggttctcatcaaatacag
	Antisense	ctgtatttgatgagaaccgaatctggtacctcacagag
S584G (AGC>AGG)	Sense	gtgaggtaccagcctcggttctcatcaaatacagaaaa
	Antisense	ttttctgtatttgatgagaaccgaggctggtacctcac
W585R (TGG>CGG)	Sense	ttgatgagaaccgaagccggtacctcacagagaat
	Antisense	aactactcttggcttcggccatggagtgtctctta
W585C (TGG>TTG)	Sense	gatgagaaccgaagctgctacctcacagagaatat
	Antisense	ctactcttggcttcgacgatggagtgtctcttata
W585L (TGG-TGC)	Sense	atttgatgagaaccgaagcttgtacctcacagagaatatac
	Antisense	taaactactcttggcttcgaacatggagtgtctcttatatg
D580H (GAT>CAT) +N582D (AAC>GAC)	Sense	gttttctgtatttcatgaggaccgaagctggtacctca
	Antisense	tgaggtaccagcttcggtcctcatgaaatacagaaaac
S584T (AGC>ACC) +N582D (AAC>GAC)	Sense	gttttctgtatttgatgaggaccgaacctggtacctca
	Antisense	tgaggtaccaggttcggtcctcatcaaatacagaaaac
I566T (ATA>ACA)	Sense	cattcctcttgtctgacattgtctggtttcctctttgatct
	Antisense	agatcaaagaggaaaccagacaatgtcagacaagaggaatg

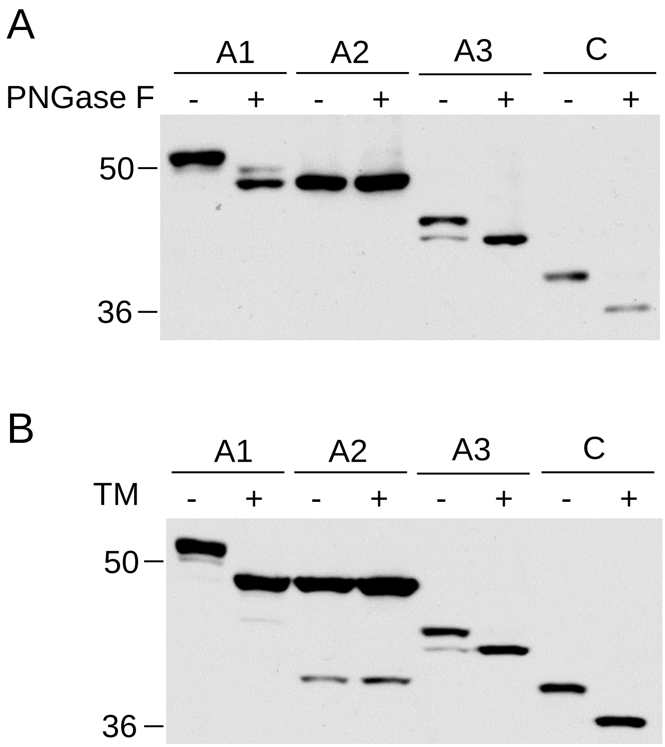

Figure S1. Glycosylation status of isolated FVIII domains expressed in HEK293T cells. (A) HEK293T cells were transfected with constructs that express Flag-tagged individual domains (A1, A2, A3 and C) of FVIII. Cell lysates were treated with or without PNGase F digestion, and subject to immunoblotting with an anti-Flag antibody. (B) HEK293T cells were transfected with individual FVIII domain constructs in duplicates. One set of transfected cells were treated with 2 μg/ml tunicamycin for 12 h before lysis.

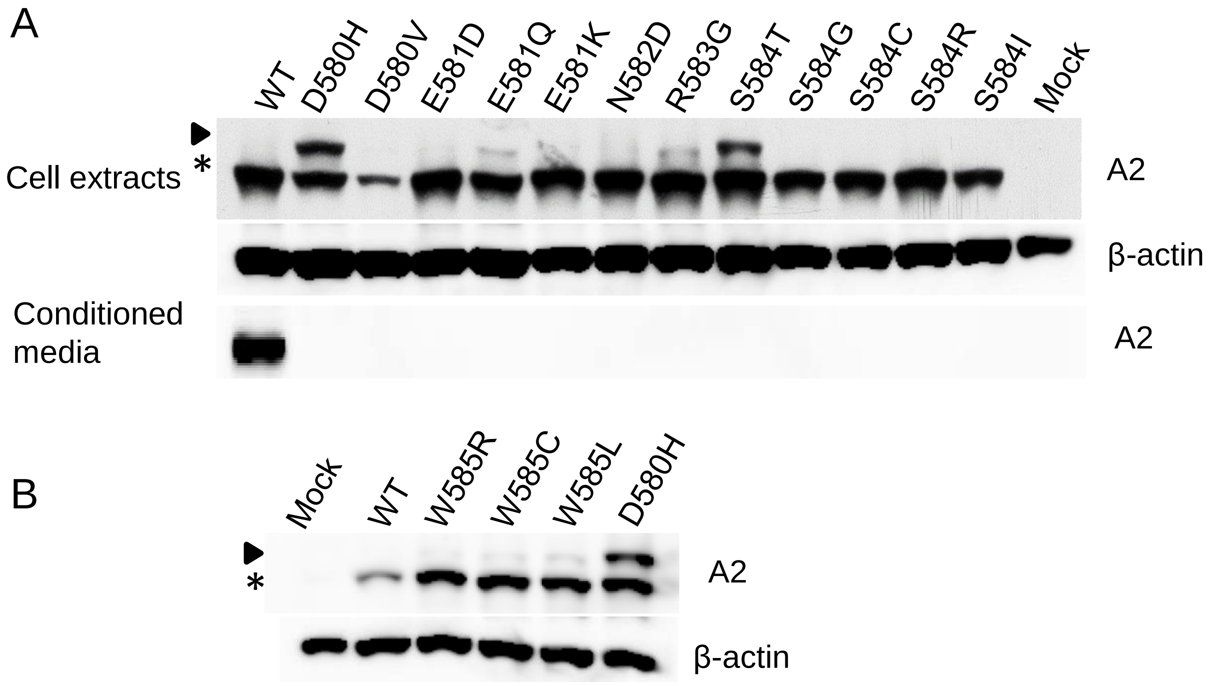

Figure S2. Glycosylation status of the A2 domain with missense mutations adjacent to N601 (D599 to S604) expressedmin HEK293T cells. (A) Extracts of HEK293T cells transiently transfected with constructs expressing WT A2 and the indicated A2 mutants were collected at 36 h after transfection and analyzed by 10% SDS-PAGE and immunoblotting. Arrowhead indicates glycosylated A2 domain and asterisk indicates non-glycosylated A2 domain. Protein levels in the media were detected by immunoprecipitation with an anti-Flag antibody followed by immunoblotting. (B) Extracts of HEK293T cells transiently transfected with constructs expressing WT A2 and the indicated A2 mutants were collected at 36 h after transfection and analyzed by 10% SDS-PAGE and immunoblotting. Arrowhead denotes N-glycosylated species and asterisk denotes non-glycosylated species of the A2 domain. Representative images of two independent experiments are shown.

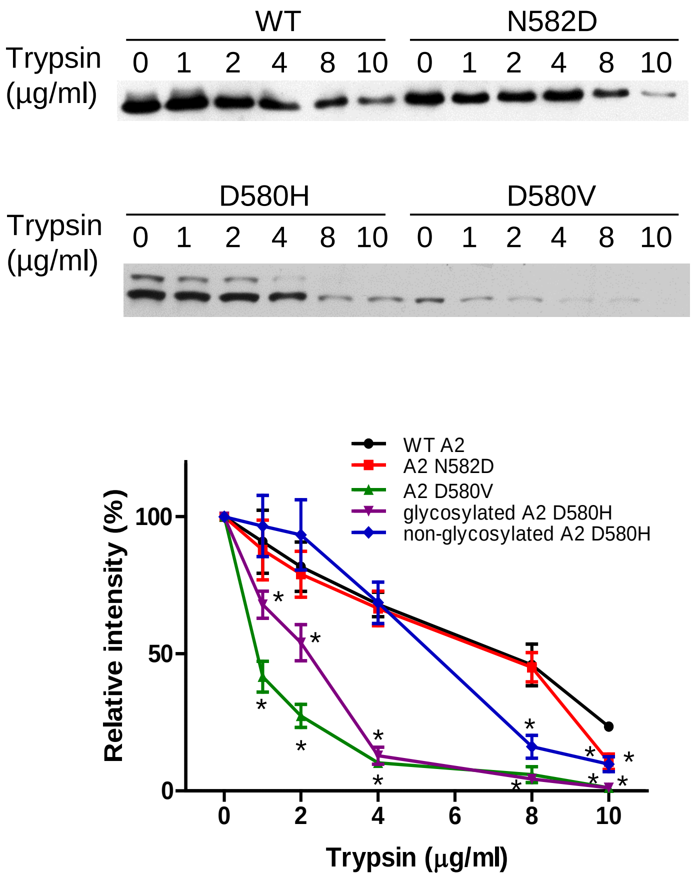

Figure S3. Protease digestion of WT and mutant FVIII A2 domain at 37 ℃. (A) COS1 cells were suspended in phosphate buffered saline and lysed by passing through a ball bearing homogenizer with 18 micron clearance (Isobiotec, Heidelberg, Germany) 36 h after transfection with the WT A2, A2-N582D, A2-D580H or A2-D580V plasmids. After removing debris by centrifugation (15,000 *g*, 10 min, 4 °C), 10 μg protein was subject to digestion by various concentrations of trypsin (0-10 μg/ml) in 15 μl reaction volume at 37 ℃ for 15 minutes. (B) The relative protein levels were quantified and plotted as percentages remaining (data are mean ± SEM, n=3. **P*<0.05).
